# NF-κB and Human Cancer: What Have We Learned over the Past 35 Years?

**DOI:** 10.3390/biomedicines9080889

**Published:** 2021-07-25

**Authors:** Thomas D. Gilmore

**Affiliations:** Biology Department, Boston University, Boston, MA 02215, USA; gilmore@bu.edu

**Keywords:** NF-kappaB, cancer, therapy, immunity, signal transduction

## Abstract

Transcription factor NF-κB has been extensively studied for its varied roles in cancer development since its initial characterization as a potent retroviral oncogene. It is now clear that NF-κB also plays a major role in a large variety of human cancers, including especially ones of immune cell origin. NF-κB is generally constitutively or aberrantly activated in human cancers where it is involved. These activations can occur due to mutations in the NF-κB transcription factors themselves, in upstream regulators of NF-κB, or in pathways that impact NF-κB. In addition, NF-κB can be activated by tumor-assisting processes such as inflammation, stromal effects, and genetic or epigenetic changes in chromatin. Aberrant NF-κB activity can affect many tumor-associated processes, including cell survival, cell cycle progression, inflammation, metastasis, angiogenesis, and regulatory T cell function. As such, inhibition of NF-κB has often been investigated as an anticancer strategy. Nevertheless, with a few exceptions, NF-κB inhibition has had limited success in human cancer treatment. This review covers general themes that have emerged regarding the biological roles and mechanisms by which NF-κB contributes to human cancers and new thoughts on how NF-κB may be targeted for cancer prognosis or therapy.

## 1. Introduction

Eukaryotic transcription factor NF-κB (nuclear factor kappa-light-chain-enhancer of activated B cells) has been the subject of intense study over the past 35 years for its role in a variety of normal and pathological processes [1]. Indeed, there are currently approximately 100,000 publications with information related to NF-κB. After providing some basic information on NF-κB protein structure and signaling, this review features a historical and conceptual overview of NF-κB signaling and human cancer, as well as therapeutic implications. This review is meant to serve as a broad introduction to more specific articles on NF-κB and cancer which are included in this dedicated issue of *Biomedicines.*

### 1.1. Structures of NF-κB and IκB Proteins

In humans, the NF-κB superfamily comprises five transcription factors: REL (also known as c-Rel), RELA (also known as p65), RELB, NFKB1 (p105), and NFKB2 (p100) [2]. These proteins are related on the basis of a conserved N-terminal domain called the Rel homology domain (RHD), which contains residues important for DNA binding, dimerization, and nuclear localization. Based on the extent of amino acid sequence similarity within the RHD and protein domains C-terminal to the RHD, the NF-κB superfamily is usually divided into two subfamilies: the Rel proteins (REL, RELA, RELB) and the NF-κB proteins (p100 and p105). The Rel family proteins have C-terminal transactivation domains, whereas the NF-κB proteins have C-terminal ankyrin (ANK) repeat domains. For the most part, all five NF-κB superfamily proteins can form all combinations of heterodimers, as well as homodimers. Although generally activators of transcription, in some situations, NF-κB dimers repress transcription of target genes. Whether a given NF-κB dimer can activate or repress transcription can depend on the DNA site it binds to or its interaction with different cofactors or other transcription factors at a given transcriptional regulatory site [3]. Moreover, NF-κB transcription factors can interact with DNA regulatory elements indirectly due to interactions with other transcription factors that are bound to DNA [4].

**Figure 1:**
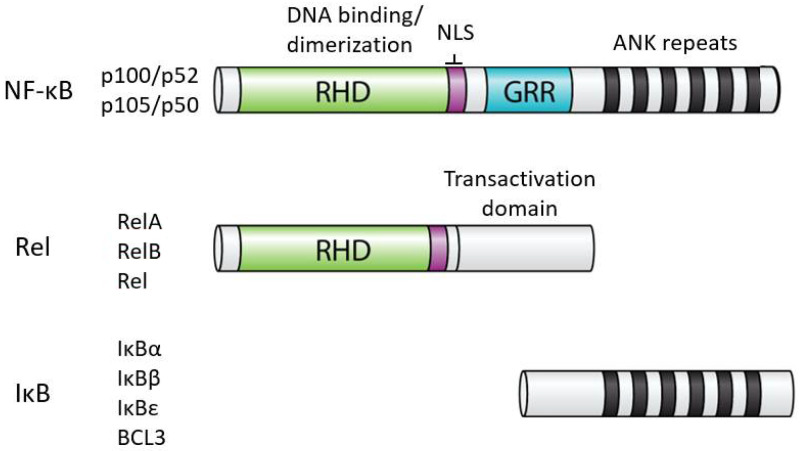
General structures of NF-κB and IκB proteins. Shown are the general structures of human NF-κB and Rel proteins, as well as IκB proteins (i.e., IκBα, IκBβ, IκBε, BCL3). RHD, Rel homology domain; NLS, nuclear localization sequence; GRR, glycine-rich region; ANK, ankyrin repeats (black bars). The details of the indicated protein domains are described in the text.

The activity of all NF-κB dimers is modulated by interaction with a family of NF-κB inhibitor proteins called IκBs [5]. All IκB proteins consist of a series of 5–8 ANK repeats, which are protein interaction domains that interact with the RHD sequences and block the ability of NF-κB dimers to bind to DNA and translocate to the nucleus. There are several independent IκB proteins (IκBα, -β, -ε, BCL3) which have different cell type-specific expression and have different affinities for the different NF-κB dimers. In addition, the C-terminal sequences of p100 and p105 contain ANK repeat IκB sequences that interact intramolecularly with the RHD sequences to block their activity. The general structures of NF-κB and IκB proteins are shown in Figure 1.

### 1.2. Activation of NF-κB Dimers Involves Nuclear Translocation

**Figure 2:**
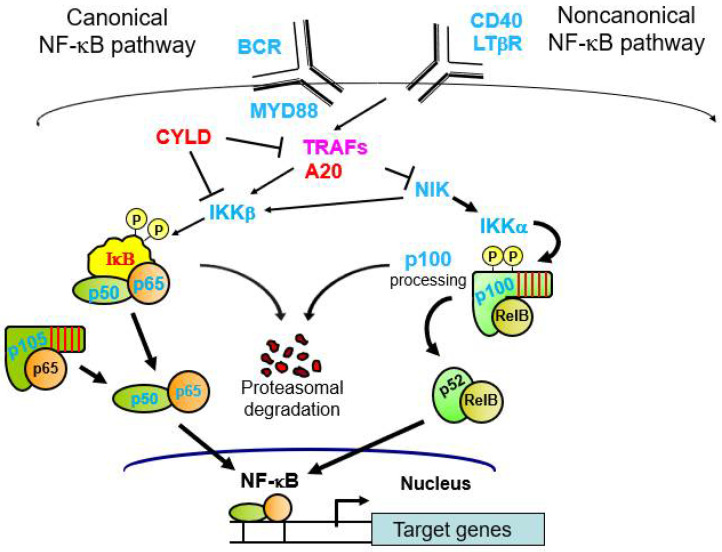
Canonical and noncanonical NF-κB pathways. Shown are the two most common NF-κB pathways, including several of the pathway components that are affected by mutations in cancer. Blue font, affected by gain-of-function mutations; red font, affected by loss-of-function mutations; purple font, affected by both gain- and loss-of-function mutations. See text for more details.

In most basal situations, NF-κB–IκB complexes are located in the cytoplasm [5]. Activation of NF-κB involves signal-induced degradation of the IκB protein, which frees NF-κB to enter the nucleus to affect gene transcription. The primary regulators of IκB stability are two kinases known as IκB kinases (IKK) α and β. These two kinases regulate two primary NF-κB activation pathways called the canonical and noncanonical NF-κB pathways (Figure 2). Thus, the core NF-κB signaling pathway consists of NF-κB dimers, IκB proteins, and IKKs.

In the canonical pathway, IκBα bound to an NF-κB dimer (e.g., p50–RELA, p50–REL) is phosphorylated by IKKβ, which induces ubiquitination and proteasome-mediated degradation of IκBα [5]. In the noncanonical pathway, a p100–RELB dimer undergoes induced degradation of the p100 C-terminal sequences to become p52–RELB [6]. That is, following IKKα-mediated phosphorylation of serine residues in the C-terminal half of p100, there is proteasomal processing of the ANK repeats to generate p52 from p100. In both the canonical and noncanonical pathways, the NF-κB dimer (e.g., p50–RELA or p52–RELB, respectively) can enter the nucleus and bind to DNA. There are many subtle differences in NF-κB signaling and activation in different cell types and depending on the given NF-κB–IκB complex involved, but those details are beyond the scope of this review.

### 1.3. Upstream Pathways That Lead to Activation of NF-κB

There are over 300 different signals that are now known to activate IKK proteins to cause downstream activation of NF-κB (see nf-kb.org). In many cases, the ultimate upstream activators are receptor-like proteins that bind to ligands [5]. Ligand binding to the receptor then initiates a multicomponent signaling pathway that leads to activation of a given IKK complex and a downstream NF-κB pathway. 

### 1.4. Signal-Induced Activation of NF-κB

Two types of well-studied upstream activators of canonical NF-κB signaling are cytokine receptors (e.g., tumor necrosis factor (TNF), interferons, interleukins (ILs)) [7] and pathogen receptors (e.g., Toll-like receptors (TLRs) or cGAS–STING complexes) [8,9,10]. The most common activators of noncanonical NF-κB signaling are the B cell activating factor (BAFF), the cell surface receptor CD40, and the lymphotoxin-β receptor [6]. Nevertheless, there is much overlap between the two pathways, and several ligands that induce activation of NF-κB can activate both the canonical and noncanonical pathways, even in the same cell type [6]. In almost all cases, cancer-related effects on NF-κB involve an alteration that causes chronic activation of nuclear canonical or noncanonical NF-κB complexes. 

### 1.5. Overview of NF-κB and Cancer

The first evidence for a role of NF-κB in cancer came from the characterization of the v-*rel* oncogene of the avian Rev-T retrovirus [11]. Indeed, Rev-T is an extremely potent oncogenic agent and causes a rapidly lethal lymphoma when injected into young chickens. Moreover, substitution of the human *REL* proto-oncogene for v-*rel* in Rev-T is also highly oncogenic in chicken lymphoid cells, both in vivo and in vitro. 

In spite of its potency as a single-agent oncogene in avian systems, NF-κB has not emerged as an efficacious mammalian oncogene in the manner of RAS, MYC, or SRC. Namely, no NF-κB protein has been shown to have rapid and strong oncogenic activity in any mammalian cell line or transgenic mouse model. Nevertheless, constitutively active canonical and noncanonical NF-κB has been found in over 40 cancer types (Table 1), and this activity has been implicated in a variety of standard cancer-associated biological processes, including cell survival, cell proliferation, metastasis, inflammation, angiogenesis, immune cell inhibition, growth factor activity, and stromal cell effects [12,13]. Relevant NF-κB target genes for many of these biological situations have been identified.

## 2. Cancer Cell-Induced Activation of NF-κB

Unlike what is found in most normal cells, NF-κB is constitutively located in the nucleus in many cancer cells. As discussed in this section, constitutive activation of NF-κB can occur by several mechanisms and in a large number of different cancers.

### 2.1. Mutational Activation of NF-κB in Cancer

NF-κB activity can be activated by cancer-associated gene mutation. These mutations can occur in both core NF-κB pathway proteins and upstream regulators of NF-κB (Table 2), and these mutations can affect either canonical or noncanonical NF-κB signaling. Such mutations are perhaps best characterized in hematological malignancies, including prominently B cell and T cell leukemias and lymphomas [14] and multiple myeloma [15]. Nevertheless, cancer genome sequencing projects are now uncovering NF-κB pathway mutations in many other cancer types.

The most highly studied malignancies with NF-κB mutations are diffuse large B cell lymphoma (DLBCL) [16] and multiple myeloma (MM) [17]. Indeed, mutation-based activation of canonical NF-κB defines a molecular subtype of DLBCL, which has a poorer clinical outcome in response to standard chemotherapy [16]. The canonical NF-κB-dependent subtype is called the activated B cell (ABC) subtype, which was first defined by similarity to an mRNA expression profile of normal antigen-activated B cells that have high expression of several direct NF-κB target genes [15]. Activating mutations of NF-κB occur in about 40% of DLBCLs and most commonly occur at multiple steps in the B cell receptor (BCR)-to-NF-κB signaling pathway [18]. Frequent activation of the noncanonical NF-κB pathway has also been reported in all DLBCL subtypes [19] regardless of the canonical pathway status, indicating that noncanonical NF-κB signaling also represents a major driver of DLBCL. In DLBCL, some gain-of-function mutations generate chronic activators of NF-κB (e.g., mutations in CD79, MYD88, or *REL* gene amplification), whereas other loss-of-function mutations inactivate negative regulators of NF-κB (e.g., IκB, CYLD, A20, TRAF3) [19,20]. Similarly, multiple pathway activators can lead to constitutive canonical and noncanonical NF-κB pathway activation in MM in 20–40% of cases [15,21]. Moreover, NF-κB pathway inhibition can block the proliferation and survival of DLBCL and MM cell lines that have chronic activation of NF-κB in a variety of in vitro cell and in vivo mouse models [15,16].

Nevertheless, it is important to note that mutation-based activation of NF-κB is simply one oncogenic driver pathway in most B cell malignancies. That is, there is a variety of other cooperating mutations in other cellular pathways, e.g., in proteins such as NOTCH, BCL6, IRF4, p300/CBP, that cluster with NF-κB pathway mutations to define further distinct genetic subtypes of DLBCL [18]. In some cases, these complementing alterations in signaling pathways may be required to keep NF-κB activity in the optimal range for the malignant process. 

#### 2.1.1. Mutation of Core NF-κB Signaling Proteins

NF-κB transcription factors are themselves targets for mutation in many cancers, although RELA and RELB are much less frequently mutated than REL, NFKB1, and NFKB2. Generally, oncogenic alterations in NF-κB proteins result in C-terminal truncations or single amino acid mutations, which lead to enhanced activity [22]. For example, C-terminal truncations that remove ANK repeat inhibitory sequences of p100 have been found in B and T cell lymphomas. C-terminal mutations that alter the transactivation activity have been characterized in REL and RELA [22]. *REL* gene amplifications, leading to increased REL protein expression, have been found in several types of lymphoma, including DLBCL and Hodgkin lymphoma [23,24]. 

Complete loss-of-function mutations have been found in IκB proteins, which lead to chronic nuclear localization of NF-κB in DLBCL [25], Hodgkin lymphoma [26], glioblastoma [27], and nasopharyngeal cancer [28]. Although much less frequent than mutations in NF-κB subunits or IκBs, constitutively activating mutations in IKKβ have been found in MM [29], mantle cell and marginal zone lymphomas [29], and prostate cancer [30], and IKKα mutations have been found in several cancer types [31]. The rarity of IKK mutations may be because IKK can function in a variety of signaling pathways in addition to NF-κB [32] and because overexpression of IKK is toxic in several cell systems.

#### 2.1.2. Mutation of Upstream Regulators of NF-κB in Lymphoma/Leukemia

Probably the most common type of mutations for enhancing NF-κB signaling occurs in upstream or downstream molecules in specific pathways. In DLBCL and MM, this can include mutations in receptors (i.e., CD79 of the B cell receptor), adaptors (e.g., MYD88, CARMA1, BCL10) and pathway modulators (A20, CYLD, TRAFs, BTK, BIRC2/3) [15,16,17,18,19]. In short, DLBCL and MM are essentially genetic experiments that select for tumor cells that have mutations that drive sustained activation of canonical or noncanonical NF-κB, which is required for malignant B cell proliferation and survival. Moreover, many DLBCLs or Hodgkin lymphomas have mutations in the transcriptional coactivators CBP, p300, and BCL3 [33], which are known to interact with NF-κB subunits on DNA to drive transcription. These coactivator mutations have been proposed to bring constitutive NF-κB-driven transcription to the optimal range for the given tumor [34]. Indeed, one human DLBCL cell line has been shown to have mutations that alter the activity of three NF-κB pathway proteins (REL, IκB, p300) [24,25].

**Table 2:** Some mutations in the NF-κB pathway in cancers.

Gene	Protein	Type of Mutations	Effect on Activity	Cancer Type	Ref.
Core NF-κB pathway proteins					
*NFKB1*	p50/p105	Point mutations	Increase	CC, L, G, O	[35,36]
*NFKB2*	p52/p100	C-terminal truncations	Increase	B and T cell L/Lym	[22]
*RELA*	RELA (p65)	Point mutations	Increase	MM	[37]
*REL*	REL	Amplifications Point mutations Truncations	Increase Increase Decrease	DLBCL FL, DLBCL DLBCL	[24]
*NFKBIA*	IκBα	Mutations/deletions	Decrease	DLBCL, GB, NPC	[22,27,28]
*NFIKBB*	IκBβ	Point mutation	Decrease	MM	[38]
*NFKBIE*	IκBε	Deletions, point mutations	Decrease	CLL	[39]
*BCL3*	BCL3	Translocations	Increase	CLL	[40]
*CHUK*	IKKα	Point mutations			
*IKBKB*	IKKβ	Point mutations	Increase	Pr, MM, Lym	[29,30]
*MAP3K14*	NIK	Gene fusion, point mutations	Increase	MM, HL	[38,41]
Upstream modulators					
*CD79A/B*	CD79A/B	Point mutations	Increase	Lym	[14]
*MYD88*	MYD88	Point mutations, deletions	Increase	Many	[18]
*BCL10*	BCL10	Point mutations, chromosomal translocations	Increase	Lym	[42]
*MALT1*	MALT1	Chromosomal translocations, point mutations, amplifications	Increase	Lym	[43]
*TNFAIP3*	A20	Point mutations	Decrease	Lym, NP	[28,44]
*CYLD*	CYLD	Mutations, deletions	Decrease	Cyld, NP, MM	[45]
*TRAF1,4,5,6*	TRAF1,4,5,6	Amplifications, point mutations, deletions	Increase	Many	[46]
*TRAF2,3*	TRAF2,3	Point mutation, deletion, amplifcation	Decrease	Many	[46]
*CARMA1*	CARD11	Chromosomal translocation; Point mutation	Increase	Leuk, Lym	[47]
*HOIP, HOIL, SHARPIN*	LUBAC	Point mutations	Increase	DLBCL	[48]
Coactivators					
*EP300*	p300	Deletions	Decrease	DLBCL, Leuk, ST	[34,49]
*CREBBP*	CBP	Deletions	Decrease	DLBCL, Leuk, ST	[34,49]

CC, cervical cancer; CLL, chronic lymphocytic leukemia; Cyld, cylindromatosis; DLBCL, diffuse large B cell lymphoma; G, gastric; GB, glioblastoma; HL, Hodgkin lymphoma; L, liver; Leuk, many leukemias; Lym, various B cell lymphomas; Many, many types of cancer; MM, multiple myeloma; NP, nasopharyngeal carcinoma; O, ovarian; Pr, prostate; ST, many solid tumors.

### 2.2. Chronic Signal-Induced Activation of NF-κB in Some Cancers

NF-κB shows an oscillating pattern of activation/inactivation in the presence of a continous activating signal due to the negative feedback in NF-κB signaling provided by new synthesis of IκB [50]. In many tumor settings, constitutive activation of NF-κB can also be achieved by autocrine, often stromal, activation of NF-κB (e.g., by cytokines [51] or other stimulus-dependent activations of the pathway). For example, TNF is encoded by a direct NF-κB target gene that can function as a chronic activator of NF-κB in an autocrine manner for certain tumor cells with constitutive activation of NF-κB [52]. Furthermore, some DLBCLs become dependent on TLR-based activation of NF-κB by specific antigens [18]. In other cases, chronic stimulation of the TLR pathway by pathogens has been associated with cancer. For example, Muto et al. [53] showed that *TRAF6* overexpression resulting from somatic alterations in preleukemic myelodysplastic hematopoietic stem cells (MDS HSCs) causes a switch from canonical to noncanonical NF-κB signaling during inflammation; this switch to noncanonical signaling provides these MDS HSCs with a competitive growth and survival advantage over noninflamed normal HSCs.

### 2.3. Epigenetic and Genomic Effects Involving NF-κB in Cancer

A variety of studies have shown that chromatin state can influence the binding of NF-κB to various promoters [3,54]. That is, induced NF-κB binds preferentially to genomic sites that are epigenetically primed. In addition, NF-κB can recruit chromatin-modifying coactivators (e.g., histone acetyltransferases and histone deacetylases) to further modify local chromatin. Given the broad role of epigenetic changes in cancer [55], it is not surprising that there can also be interplay between epigenetic DNA status or epigenetic regulators and the chronically active NF-κB that is found in many cancers. For example, the gene encoding the epigenetic regulator protein MLL is changed by chromosomal alterations that generate oncogenic MLL fusion proteins that drive many leukemias, and the activity of these leukemogenic MLL fusion proteins requires NF-κB [56]. On the other hand, epigenetic methylation at the promoter of the FAS death receptor in certain cancers reduces NF-κB-dependent activation of FAS expression, thus contributing to tumor cell survival by reducing levels of the proapoptotic FAS protein [57].

Similarly, it is now clear that mutations in noncoding sequences can also contribute to cancer by affecting transcription and gene expression [58]. Mutations in promoter sequences that affect *NFKB1* expression or NF-κB-dependent expression of target genes have been described in oral, bladder, and breast cancer [59,60,61]. Furthermore, aberrant alternative splicing of the NF-κB inhibitor CYLD may lead to chronic activaiton of NF-κB in some cases of chronic lymphocytic leukemia [62].

### 2.4. Oncogenic Human Viruses That Affect NF-κB Signaling

Several human viruses have been associated with cancer, and many of these viruses use cellular signaling pathways, including NF-κB, for their replication and pathogenesis [63]. Often, these viruses are oncogenic when their replication is aborted or chronic. Thus, these viruses can contribute to the oncogenic state either through chronic NF-κB-dependent inflammation or by the sustained activity of viral activators of NF-κB. Examples of virally encoded NF-κB activators include the LMP1 protein of Epstein–Barr virus (B cell lymphoma, nasopharyngeal carcinoma) [64], the vFLIP protein of Kaposi sarcoma herpesvirus (sarcoma, lymphoma) [64], the Tax protein of HTLV-1 (T cell leukemia) [65], the X protein of hepatitis B virus (liver cancer) [66], and the E6 and E7 proteins of some strains of the human papillomavirus (cervical cancer) [67].

## 3. Cancer-Related Processes Affected by Chronic Activation of NF-κB

### 3.1. Cell Survival

One of the many cancer-promoting cellular processes affected by NF-κB is cell survival, where NF-κB generally acts by blocking apoptosis [68]. The first experiments demonstrating a role for NF-κB in cell survival were ones showing that inactivation of the v-*rel* oncogene caused transformed lymphoid cells to die of apoptosis [69] and that knockout of the *rela* gene caused mouse fibroblasts and embryonic liver cells to undergo apoptosis in response to the tumor necrosis factor [70,71]. Thus, in a variety of tumor cells with constitutive NF-κB activity, one finds upregulation of direct NF-κB target genes that encode antiapoptotic molecules such as BCL2, BCLXL, and IAPs. In particular, NF-κB’s ability to block apoptosis is important for B and T cell malignancies where their normal endpoint of proliferation cycles is apoptosis [16] and in the resistance of tumor cells to chemotherapeutic agents and radiation [72].

In some cancer-related situations, however, NF-κB is required for apoptosis. In most of these cases, NF-κB is required for efficient drug-induced apoptosis in various cancer cell lines. For example, inhibition of NF-κB reduces apoptosis induced by doxorubicin in neuroblastoma cells [73], by etoposide in HL-60 myeloid leukemia cells [74], and by UV irradiation in melanoma cells [75]. In some of these cases, NF-κB may be a cell type-specific repressor of the antiapoptotic genes discussed above or an activator of proapoptotic genes such as BAX [76], BIM [77], CASPASE-11 [78], and FAS [79].

### 3.2. Cell Proliferation

Several NF-κB target genes that contribute to cell proliferation have been identified. Such direct pro-proliferation targets include several cell cycle genes [80], including notably cyclin D1 [81], and the CD44 gene required for breast cancer cell proliferation [82]. Furthermore, in several settings, NF-κB has been shown to be a required downstream pathway for RAS-induced transformation [83,84]. It is not always clear whether the pro-proliferative dysregulation of NF-κB is directly involved in changes in expression of proliferation genes or it is due to a change in the apoptotic or epigenetic state of the tumor cell. 

### 3.3. Inflammation

NF-κB plays a prominent role in innate immunity in both normal inflammation and dysregulated chronic disease-promoting inflammation that is associated with autoimmune diseases, tissue damage, and many types of cancer [85]. Chronic inflammation contributes to cancer by increasing cellular stress responses, recruiting inflammatory factors, and can also change the epigenetic state of many genes. As such, many cytokine genes (e.g., TNFα, IL-1, IL-6, IL-17) are direct targets of NF-κB and are known to be protumorigenic, especially as related to stomach/bowel cancers (e.g., inflammatory bowel disease) [86,87] and stromal contributions in MM [16]. Intriguingly, one recent large-scale study [4] showed that many cancers have an inflammatory gene network that involves interactions of the transcription factors STAT3, NF-κB, and AP-1 at common DNA sites where NF-κB, in many cases, is only indirectly associated with DNA by virtue of its interaction with STAT3 or AP-1 on DNA. 

### 3.4. Other Hallmarks of Cancer: Angiogenesis, Metastasis, and Cell Immortalization

In addition to the direct effects of inflammatory molecules on cancer cell properties such as proliferation, survival, and inflammation, cancer-activated NF-κB can promote tumor-associated processes such as angiogenesis [88], epithelial mesenchymal transition (EMT) (e.g., matrix metalloproteinase-3 (MMP3) expression [89]), and metastasis (e.g., MMP9 regulation [90]). 

Several proangiogenic molecules, including VEGF (vascular endothelial growth factor), IL-8, and MMP9, have been shown to be downregulated in prostate cancer and hemangioma tumor cells [91], and inhibition of NF-κB in these cells blocked their ability to promote angiogenesis. Furthermore, NF-κB has been shown to be important for blood vessel endothelial cell survival in response to TNF [92], which is present at high levels in many inflamed cancers. Several of the same types of molecules that play a role in angiogenesis also promote EMT and metastasis. Thus, it is not surprising that NF-κB has also been shown to have a role in some metastasis models, including pancreatic cancer [93]. Moreover, some of the primary EMT molecules, including TWIST, SLUG, and SIP1, have been shown to be regulated by NF-κB [94].

NF-κB has also been implicated in cellular immortalization. For example, NF-κB has been shown to have a complex interaction with the catalytic subunit of telomerase (hTERT): that is, the *hTERT* gene has been reported to be a direct target for NF-κB [95] and the hTERT protein has been shown to interact directly with RELA to affect RELA’s nuclear translocation and transcriptional activation activity [89]. Similarly, NF-κB has been reported to induce the bypass of senescence in melanoma cells [96].

### 3.5. Immunosuppression

In the past decade, it has become clear that many tumors create an immunosuppressed environment that is required for their progression. In some cases, this immunosuppression involves the recruitment of immune cells such as regulatory T cells (Tregs) or M2-biased tumor-infiltrating macrophages (TAMs) to the tumor. NF-κB has been shown to be involved in these processes in several ways. First, c-*rel* is required for the development of Tregs [97]. Second, increased NF-κB p50 activity is associated with an M1- to M2-polarized (protumorigenic and immunosuppressive) state in macrophages [98,99]. In addition, the gene encoding the checkpoint inhibitor protein PD-L1 is an NF-κB target gene [100], suggesting that inflammation can promote a T cell-based immunosuppressive environment that contributes to the survival of some tumors.

Thus, NF-κB can contribute directly to tumor cell proliferation and survival, as well as to biological processes that affect the malignant state and progression of the tumor. Cancer-related biological processes regulated by NF-κB and some of the NF-κB target genes involved in these processes are listed in Table 3.

## 4. NF-κB as a Tumor Suppressor

In a limited number of cases, NF-κB has been reported to have tumor suppressor activity. However, this activity has generally been demonstrated in mouse or cell-based models. For example, downregulation of NF-κB by transgenic overexpression of IκBα led to skin epidermal hyperplasia in mice [108], and some RelA knockout mouse cell lines have a weakly transformed phenotype [109]. However, in both of those cases, the direct oncogenic effect of reduced NF-κB activity is not clear. Moreover, tumor suppressor activity of NF-κB has not been well-documented in human cancers [110]. Nevertheless, the extensive crosstalk between NF-κB and both wild-type and mutant tumor suppressor p53 indicates that NF-κB may play a role in tumor suppression in humans in certain circumstances [111].

## 5. Targeting of NF-κB for Cancer Therapy

Based on the pervasive involvement of NF-κB in oncogenesis, it is not surprising that NF-κB signaling would be considered as a target for human cancer therapy. Indeed, there are well over 1000 inhibitors that can block NF-κB signaling [112], and inhibition of NF-κB can block cancer cell proliferation or survival in a number of animal models. Nevertheless, inhibitors of NF-κB signaling have not had a dramatic impact on general cancer therapy, in part because of the liver toxicity of many NF-κB inhibitors and the rapid development of parallel pathway resistance. Still, proteasome inhibitors (which block IκB degradation for canonical and noncanonical signaling) have been quite effective for the treatment of multiple myeloma [15], and they appear to act, at least in part, due to their inhibitory effects on NF-κB signaling. Similarly, Bruton’s tyrosine kinase (BTK) inhibitors have been useful in the treatment of NF-κB-positive DLBCL [113] as BTK is required for the BCR–NF-κB pathway in B cells. Finally, whether inhibition of NF-κB might promote cancer in some cases by affecting tumor-suppressing activity of NF-κB or by having a detrimental effect on patient antitumor T cell immunity (natural or induced by immunotherapy) is not clear.

## 6. Future Considerations

It is likely that NF-κB will be found to be associated with many more cancers, especially as we accumulate additional whole genome sequence data. Nevertheless, it is unlikely that sustained, systemic, and complete inhibition of upstream NF-κB pathways will be a useful primary strategy for cancer or any other disease. That being said, agents and doses that reduce (but do not eliminate) cancer-induced NF-κB activity or inflammation may well be useful. Moreover, it has been generally difficult to directly target the NF-κB transcription factors themselves [114], which may be a more proximal and reduced off-target strategy given, for example, the viability of c-*rel* knockout mice [24]. Still, acute and strong inhibition of NF-κB as adjuvant therapy may be useful in certain cancer regimens. Alternatively, therapies that can distinguish and target oncogenic NF-κB activity versus normal NF-κB activity are required. Indeed, direct inhibitors of oncogenic RAS have only recently started being used in the clinic for cancer therapy [115], forty years after the discovery of RAS, demonstrating the difficulty of developing useful cancer therapeutics in pervasive and important signaling pathways. Intriguingly, NF-κB has been shown to be required for downstream RAS signaling in some cancers [81,82]. Thus, it is almost certain that research on pathological NF-κB activity will continue to occupy an important place in cancer research, and modern genome modification methods and/or new therapeutic strategies to target NF-κB activity will prove useful in certain settings.

## Figures and Tables

**Table 1:** Cancers that have been reported to have high NF-κB activity.

Hematological Malignancies	Solid Tumors
Hodgkin lymphoma	Breast
Acute lymphoblastic leukemia	Cervical
Acute myelogenous leukemia	Ovarian
Acute T cell leukemia	Vulvar
Acute nonlymphocytic leukemia	Uterine (endometrial)
Chronic lymphocytic leukemia	Prostate
Chronic myelogenous leukemia	Testicular
Burkitt lymphoma (EBV)	Penile
Mantle cell lymphoma	Kidney
Myelodysplastic syndrome	Bladder
Multiple myeloma	Lung
Diffuse large B cell lymphoma	Mesothelioma
MALT lymphoma	Esophageal
Mantle cell lymphoma	Laryngeal
Marginal zone lymphoma	Liver
Waldenstrom’s macroglobulinemia	Pancreatic
	Stomach
	Colon
	Thyroid
	Parathyroid
	Melanoma
	Squamous cell carcinoma
	Head and neck
	Cylindromatosis
	Trichoepithelioma
	Hilar cholangiocarcinoma
	Oral carcinoma
	Tongue
	Astrocytoma
	GIST

For details, see http://www.bu.edu/nf-kb/physiological-mediators/diseases/.

**Table 3:** Some cancer-relevant biological processes and relevant target genes controlled by NF-κB.

Biological Process	Relevant Target Genes	Ref.
Cell survival (antiapoptosis)	BCL2, BCLXL, IAPs	[68]
Cell cycle	CCND1, CD44, CDK2, CDKN1A	[80]
Metastasis	MMP2, MMP9, UPA, SOX9	[101]
Angiogenesis	IL-1β, IL-8, MMP9, VEGF-A, FASL	[102]
Epithelial-to-mesenchymal transition	TWIST1, SLUG, SIP1, SNAIL	[94,103]
Inflammation	CXCL8, IL-6, TNFA, IL-1β, iNOS, COX2	[85]
Immortalization	TERT, EZH2	[104]
Senescence	IL-6, TNFA, IL-1β	[105]
Energy metabolism	GLUT3, SCO2	[106]
T cell inhibition	FOXP3, PDCD1	[107]
Cancer stem cells	NANOG, SOX2, CD44, KLF4	[101]
